# Identification of High Risk Carotid Artery Stenosis: A Multimodal Vascular and Perfusion Imaging Study

**DOI:** 10.3389/fneur.2019.00765

**Published:** 2019-07-16

**Authors:** Moo-Seok Park, Soonwook Kwon, Mi Ji Lee, Keon Ha Kim, Pyoung Jeon, Yang-Jin Park, Dong-Ik Kim, Young-Wook Kim, Oh Young Bang, Chin-Sang Chung, Kwang Ho Lee, Gyeong-Moon Kim

**Affiliations:** ^1^Department of Neurology, Samsung Medical Center, Sungkyunkwan University School of Medicine, Seoul, South Korea; ^2^Department of Radiology, Samsung Medical Center, Sungkyunkwan University School of Medicine, Seoul, South Korea; ^3^Department of Surgery, Samsung Medical Center, Sungkyunkwan University School of Medicine, Seoul, South Korea

**Keywords:** asymptomatic carotid artery stenosis, symptomatic carotid artery stenosis, risk factors, multimodal imaging, perfusion-weighted MRI

## Abstract

**Background:** Risk stratification of asymptomatic carotid artery stenosis (ACAS) is still an issue for carotid revascularization. We sought to identify factors associated with symptomatic carotid artery stenosis (SCAS) using multimodal imaging techniques.

**Methods:** We retrospectively collected data on patients who underwent carotid artery revascularization. Results from duplex sonography, computerized tomography angiography, brain magnetic resonance imaging (MRI), magnetic resonance angiography (MRA), perfusion-weighted imaging, and demographic profiles were compared between ACAS and SCAS patients. Differences in baseline characteristics between the two groups were balanced by the propensity matching score method. Multivariable regression analysis was performed to identify factors associated with symptomaticity of carotid artery stenosis. We compared the strength of associations between significant imaging factors and symptomatic carotid stenosis using C statistics.

**Results:** A total of 259 patients (asymptomatic 57.1%, symptomatic 42.9%) with carotid stenosis were included. After 1:1 propensity score matching, the multivariable regression analysis revealed that the absence of plaque calcification [Odds ratio 0.41, 95% confidence interval (CI) 0.182–0.870, *p* = 0.023], deep white matter hyperintensity (DWMH; Odds ratio 3.46, 95% CI 1.842–6.682, *p* < 0.001), susceptibility vessel sign seen on gradient-echo MRI (Odds ratio 2.35, 95% CI 1.113–5.107, *p* = 0.027), and increased cerebral blood volume (CBV) seen on perfusion-weighted MRI (CBV; Odds ratio 2.17, 95% CI 1.075–4.454, *p* = 0.032) were associated with SCAS. The combination of these variables had a fair accuracy to classify SCAS (Area under the curve 0.733, 95% CI 0.662–0.803).

**Conclusions:** We identified several multimodal imaging markers independently associated with SCAS. These markers may provide information to identify ACAS patients with high risk of ischemic stroke. Future studies are needed to predict SCAS using our findings in other independent cohorts.

## Background

Carotid artery stenosis is one of the major causes of stroke. In the population over 65 years old, 5–10% of people have over a 50%-degree of stenosis in the proximal carotid artery and 0.3–2.0% of patients with asymptomatic carotid artery stenosis (ACAS) have an ischemic stroke event each year (1). The mechanisms of stroke or transient ischemic attack in proximal carotid stenosis are artery-to-artery embolism from an unstable plaque or hypo-perfusion due to severe stenosis with poor collaterals. Unfortunately, only 15% of patients with ACAS have warning signs before an ischemic stroke (2, 3).

Therefore, imaging predictors of ischemic stroke should be identified to identify high-risk patients with ACAS and make a clinical decision on the necessity of preventive revascularization.

We hypothesized that atherosclerotic plaque characteristics, white matter hyperintensities, and perfusion abnormalities would be associated with symptomatic carotid stenosis. Plaque characteristics have been extensively studied. Among various plaque characteristics, stenosis degree has been frequently reported to one of the risk factors. Previous studies suggested high-degree stenosis is more associated with symptomatic carotid artery stenosis (SCAS) (4, 5). However, recent studies did not reveal any significant relationship between stenosis severity and risk of stroke (2, 3). An echolucent plaque, plaque ulceration, thinning of the fibrous cap, intraplaque hemorrhage, length of stenosis, and microembolic signals are also suggested as risk factors of SCAS (6). In contrast, perfusion status has been relatively less investigated. Ipsilateral hypoperfusion has been recently highlighted as a risk factor of SCAS (7). However, clinical decisions cannot be made based on a single variable, and it is unknown which factor is more important. In addition, we questioned how to better predict symptomatic carotid stenosis using a combination of already known factors.

In this study, we attempt to identify factors associated with SCAS using multimodal imaging techniques. Plaque characteristics from ultrasonography and angiography and various perfusion markers from brain magnetic resonance imaging (MRI) and perfusion weighted imaging (PWI) were comprehensively investigated and compared between ACAS and SCAS. We aimed to identify independent imaging markers of SCAS, which may suggest the risk of impending stroke when present in ACAS, and test if the multimodal approach (i.e., combination of different imaging markers) would be superior to each method.

## Methods

### Patients

We retrospectively collected data on patients who had carotid artery revascularization procedures (carotid endarterectomy or carotid artery stenting) from April 2007 to April 2016. The decision to use carotid endarterectomy or carotid artery stenting was determined by a consensus meeting based on published guidelines (8–10). Patients who had a cerebral ischemic stroke event during the 6 months before carotid endarterectomy or carotid artery stenting were categorized in the SCAS group. The other patients were categorized in the ACAS group. Only patients with atherosclerosis in the carotid artery were included, and individuals were excluded if they had a diagnosis of aneurysm or dissection of the carotid artery, fibromuscular dysplasia, Takayasu's arteritis, or post-radiotherapy. This study was approved by Institutional Review Board of Samsung Medical Center, Seoul, Korea. (IRB No. 2018-08-059) A detailed clinical chart review was performed, and the abstracted information was recorded on a standardized data collection form. The following variables were collected: sex, age, body mass index, history of smoking, presence of hyperlipidemia, diabetes mellitus, ischemic heart disease, atrial fibrillation and peripheral artery occlusive disease, and medications such as anti-platelets, anticoagulants, or statins.

### Laboratory Data

Because dyslipidemia and hyperglycemia are well-known risk factors for atherosclerosis (11, 12), blood tests for lipid profile and blood sugar were given to all patients. Data on triglyceride, total cholesterol, low-density lipoprotein-cholesterol, high-density lipoprotein-cholesterol, fasting blood sugar, and lipoprotein A levels were collected. Echocardiography was performed in most patients. The ejection fraction (13) was obtained from echocardiography.

### Imaging Data

**Figure 1:**
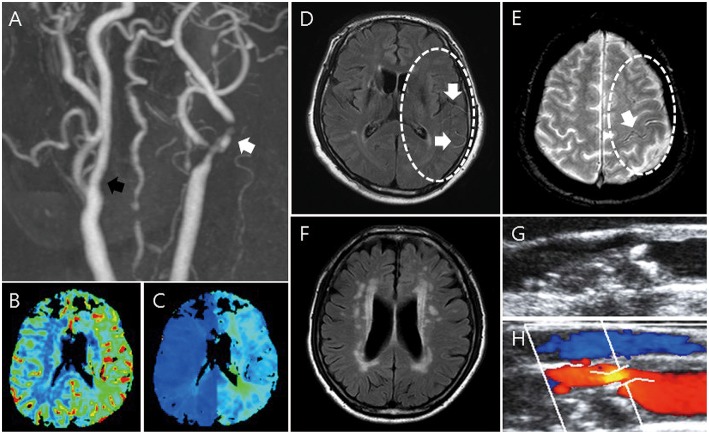
Imaging parameters: MRI, CTA/MRA, PWI, and carotid duplex sonography. MRA shows stenosis of the proximal internal carotid artery (**A**, white arrow) and contralateral carotid artery stenosis (**A**, black arrow). In PWI, increased CBV **(B)** and delayed TTP **(C)** are shown. FLAIR shows HVS in FLAIR **(D)** and PWMH/DWMH **(F)**. GRE shows HV in GRE **(E)**. Through duplex carotid sonography, plaque characteristics such as calcification, ulceration, echolucent, and stenosis are shown **(G,H)**.

Carotid duplex sonography, computed tomography angiography, MRI, magnetic resonance angiography (MRA) and PWI were performed (Figure 1). Several parameters presenting carotid plaque pathology (14) are obtained using Carotid Doppler ultrasound machine (Acuson Antares, Siemens, Munich, Germany) with a 7.5-MHz linear array probe. In carotid duplex sonography, the degree, and velocity of stenosis site and plaque nature (calcification, ulceration, and echogenicity) were recorded. The angiography values were determined from computed tomography angiography and MRA. The length of the plaque in angiography was measured from the normal to other normal side. The degree of stenosis (≥70%) and presence of contralateral carotid stenosis, ipsilateral tandem lesion, and ulceration were recorded.

MRI was performed using a 3.0-T unit (Achieva; Philips Medical Systems). The typical stroke MRI protocol included at least DWI, perfusion-weighted imaging, FLAIR, and vascular images (3-dimensional time-of-flight MRA and contrast-enhanced MRA including the extracranial ICA and the vertebral artery; Figure 1). From brain MRI, the presence of deep or periventricular white matter hyperintensity (DWMH or PWMH) (15), hyperintense vessel signal (HVS) in FLAIR (16, 17), and hypointense vessels (HV) in GRE (18) were obtained by comparing to the contralateral side. On PWI, increased relative cerebral blood volume (CBV) and delayed time to peak (TTP) were evaluated by comparing with the contralateral side and posterior circulation.

### Statistics

To compare the two groups (ACAS and SCAS groups), chi-square tests and independent Student *t*-tests were performed for dichotomous variables and continuous variables, respectively. Propensity score matching was performed to adjust for differences in baseline characteristics between ACAS and SCAS groups. Propensity scores were generated using the following variables: age, sex, body mass index, the type of procedure (carotid endarterectomy or stenting), the side of stenosis, smoking, hypertension, diabetes, hyperlipidemia, Ischemic heart disease, previous CABG or PCI, peripheral artery occlusive disease, atrial fibrillation, congestive heart failure, use of antiplatelet drug, anticoagulant, and statin, and the dose of statin. After propensity score generation, patients underwent 1:1 nearest neighbor (Greedy-type) matching of the logit of the propensity score with a caliper width of 0.25. We performed the univariable logistic regression analysis and included and adjusted variables with univariable *p* < 0.10 in the multivariable logistic regression model. Variables were selected after checking for multicollinearity using the correlation coefficient and variance inflation factor (VIF). Variables with a correlation coefficient above 0.3 with statistical significance or VIF > 10 were considered to have multicollinearity and excluded from the multivariable logistic regression analyses.

The receiver operating characteristic (ROC) curves were generated using various combination of imaging factors independently associated with SCAS. The area under ROC (AUC) was calculated to measure the accuracy of each combination to differentiated SCAS from ACAS (19). Statistical analyses were performed with R software (R version 3.2.5, The R Foundation for Statistical Computing).

## Results

### Patient Characteristics

From the total 1,096 patients during the study period, 612 patients without imaging data, 33 patients without echocardiography, 122 patients without carotid sonography, and 70 patients with incomplete laboratory data were excluded. Finally, 259 patients who had complete multimodal imaging data were included in the study, and 148 (57.1%) of these patients had ACAS while the rest (42.9%) had SCAS (Figure S1).

In the unmatched data, patients with ACAS were younger than those with SCAS (*p* = 0.037). Ischemic heart disease was more frequent in the ACAS group, CABG or PCI were also more often performed in the ACAS group (40.5 vs. 26.1%, *p* = 0.022; 34.5 vs. 18.0%, *p* = 0.005, respectively). The two groups did not differ in sex, body mass index, smoking history, hyperlipidemia, diabetes mellitus, hypertension, peripheral artery occlusive disease, atrial fibrillation, and congestive heart failure. Statin use was more common in the ACAS group (80.4 vs. 68.5%, *p* = 0.039). Usage of other medications did not show significant differences between the two groups (Table 1).

**Table 1:** Baseline characteristics of unmatched and propensity-matched subgroups.

**Variable**	**Unmatched**			**Matched**		
	**ACAS (*n* = 148)**	**SCAS (*n* = 111)**	***p* Value**	**ACAS (*n* = 95)**	**SCAS (*n* = 95)**	***p* Value**
Male, *n* (%)	129 (87.2)	91 (82.0)	0.328	78 (82.1)	80 (84.2)	0.846
Age, year, mean ± SD	68.41 ± 7.50	70.41 ± 7.71	0.037	69.48 ± 7.06	70.14 ± 7.79	0.545
BMI, kg/m^2^, mean ± SD	24.09 ± 2.70	23.69 ± 3.49	0.317	23.54 ± 2.56	23.83 ± 2.94	0.466
Carotid endarterectomy, *n* (%)	110 (74.3)	78 (70.3)	0.559	70 (73.7)	66 (69.5)	0.629
Right side stenosis, *n* (%)	74 (50.0)	52 (46.8)	0.706	53 (55.8)	45 (47.4)	0.309
Smoking, *n* (%)	41 (27.7)	28 (25.2)	0.760	23 (24.2)	24 (25.3)	>0.999
Hyperlipidemia, *n* (%)	89 (60.1)	72 (64.9)	0.517	60 (63.2)	60 (63.2)	>0.999
Diabetes mellitus, *n* (%)	65 (43.9)	54 (48.6)	0.528	40 (42.1)	45 (47.4)	0.559
Hypertension, *n* (%)	120 (81.1)	88 (79.3)	0.839	77 (81.1)	75 (78.9)	0.856
Ischemic heart disease, *n* (%)	60 (40.5)	29 (26.1)	0.022	32 (33.7)	24 (25.3)	0.265
CABG or PCI, *n* (%)	51 (34.5)	20 (18.0)	0.005	26 (27.4)	17 (17.9)	0.165
PAOD, *n* (%)	33 (22.3)	15 (13.5)	0.101	17 (17.9)	14 (14.7)	0.694
Atrial fibrillation, *n* (%)	7 (4.7)	7 (6.3)	0.781	4 (4.2)	7 (7.4)	0.534
Congestive heart failure, *n* (%)	4 (2.7)	1 (0.9)	0.395	0 (0.0)	1 (1.1)	>0.999
Use of antiplatelet drug, *n* (%)	131 (88.5)	94 (84.7)	0.473	82 (86.3)	80 (84.2)	0.837
Use of anticoagulant, *n* (%)	10 (6.8)	11 (9.9)	0.490	7 (7.4)	7 (7.4)	>0.999
Use of statin, *n* (%)	119 (80.4)	76 (68.5)	0.039	41 (43.2)	41 (43.2)	>0.999
Dose of statin, mg, mean ± SD	15.68 ± 16.38	14.37 ± 17.40	0.540	69 ± 72.6	69 ± 72.6	>0.999

*ACAS, asymptomatic carotid artery stenosis; SCAS, symptomatic carotid artery stenosis, BMI, body mass index; CABG, coronary artery bypass graft surgery; PCI, percutaneous coronary intervention; PAOD, peripheral artery occlusive disease*.

### Propensity Score Matched Analysis

After propensity score matching, 95 patients in each group were balanced for baseline variables. As a result of propensity score matching, no significant differences in baseline characteristics were noted (Table 1). Table 2 shows results from univariable analyses of laboratory, echocardiography, and multimodal imaging results. Laboratory test results and echocardiography did not differ between ACAS and SCAS (Table 2). Factors associated with SCAS with univariable *p* < 0.1 were: longer stenosis length (*p* = 0.029), all variables on brain MRI and PWI (PWMH, *p* = 0.027; DWMH, *p* < 0.001; HVS in FLAIR, *p* = 0.007; HV in GRE, *p* = 0.012; increased CBV, *p* = 0.012; delayed TTP, *p* = 0.042), absent calcification of the plaque (*p* = 0.058), ulcerative plaques *p* = 0.055), and the velocity of the stenosis (*p* = 0.077).

**Table 2:** Comparison of laboratory and imaging findings between symptomatic vs. asymptomatic carotid stenosis in propensity-matched subgroups.

	**ACAS (*n* = 95)**	**SCAS (*n* = 95)**	***p* value**
**Laboratory tests**			
Total cholesterol, mg/dl, mean ± SD	146.92 ± 38.30	149.61 ± 35.16	0.614
Fasting blood sugar, mg/dl, mean ± SD	129.34 ± 43.76	127.97 ± 53.48	0.847
Triglyceride, mg/dl, mean ± SD	137.27 ± 81.46	127.00 ± 72.31	0.359
HDL-C, mg/dl, mean ± SD	43.40 ± 12.87	43.54 ± 11.02	0.937
LDL-C, mg/dl, mean ± SD	86.47 ± 33.07	89.54 ± 29.82	0.503
Lp (a), mg/dl, mean ± SD	36.58 ± 37.78	34.31 ± 28.52	0.641
**Echocardiography**			
Ejection fraction, %, mean ± SD	62.06 ± 8.54	63.68 ± 8.85	0.199
**Carotid duplex sonography**			
Velocity of the stenosis, cm/s, mean ± SD	362.40 ± 153.73	406.88 ± 189.28	0.077
Degree of the stenosis, %, mean ± SD	74.32 ± 7.82	74.92 ± 10.48	0.655
Calcification of the plaque, *n* (%)	28 (29.5)	16 (16.8)	0.058
Ulcerative plaque, *n* (%)	19 (20.0)	29 (30.5)	0.132
Echolucent plaque, *n* (%)	6 (6.3)	12 (12.6)	0.215
Angiography^*^			
Length of the stenosis, mm, mean ± SD	17.97 ± 6.68	20.28 ± 7.78	0.029
Ulcerative plaque, *n* (%)	11 (11.6)	22 (23.2)	0.055
Degree of the stenosis (≥ 70%), *n* (%)	81 (85.3)	85 (89.5)	0.512
Stenosis of contralateral carotid artery, *n* (%)	59 (62.1)	49 (51.6)	0.187
Tandem lesion of ipsilateral carotid artery, *n* (%)	19 (20.0)	14 (14.7)	0.443
**Brain MRI**			
PWMH, *n* (%)	47 (49.5)	63 (66.3)	0.027
DWMH, *n* (%)	29 (30.5)	56 (58.9)	<0.001
HVS in FLAIR, *n* (%)	9 (9.5)	24 (25.3)	0.007
HV in GRE, *n* (%)	16 (16.8)	32 (33.7)	0.012
**Perfusion-Weighted MRI**			
Increased cerebral blood volume, *n* (%)	21 (22.1)	38 (40.0)	0.012
Delayed time to peak, *n* (%)	40 (42.1)	55 (57.9)	0.042

*HDL-C, high-density lipoprotein-cholesterol; LDL-C, low-density lipoprotein-cholesterol; Lp, (a) lipoprotein a; MRI, magnetic resonance imaging; PWMH, peripheral white matter hyperintensity; DWMH, deep white matter hyperintensity; HVS in FLAIR hyperdensity vessel signs in fluid-attenuated inversion recovery, HV in GRE hypodensity vessels in gradient echo sequences susceptibility*.

* *About 45.2% patients underwent MR angiography rather than CT angiography. The rest underwent CT angiography*.

### Factors Independently Associated With SCAS

Among variables with univariable *p* < 0.1, the length of the stenosis, ulcerative plaque, calcification of the plaque, DWMH, HV in GRE, and increased CBV were included in the multivariable model after excluding variables showing significant multicollinearity (Table 3). The absence of plaque calcification (OR 0.41, 95% CI 0.182–0.870, *p* = 0.023), more extensive DWMH (OR 3.46, 95% CI 1.842–6.682, *p* < 0.001), the presence of HV in GRE (OR 2.35, 95% CI 1.113–5.107, *p* = 0.027), and increased CBV (OR 2.17, 95% CI 1.075–4.454, *p* = 0.032) were independently associated with SCAS (Table 3).

**Table 3:** Multivariable analysis of propensity-matched subgroups.

	**Odds Ratio**	**95% CI**	***p*-value**
Length of the stenosis^a^	1.04	0.989–1.085	0.146
Ulcerative plaque^a^	1.83	0.772–4.533	0.176
DWMH^b^	3.46	1.842–6.682	<0.001
HV in GRE^b^	2.35	1.113–5.107	0.027
Absence of calcified plaque^c^	0.41	0.182–0.870	0.023
Increased cerebral blood volume^d^	2.17	1.075–4.454	0.032

a *Result of angiography*.

b *Result of brain MRI*.

c *Result of carotid duplex sonography*.

d *Result of perfusion-weighted*.

*MRI; DWMH deep white matter hyperintensity, CI, confidence interval*.

### ROC Analysis

**Figure 2:**
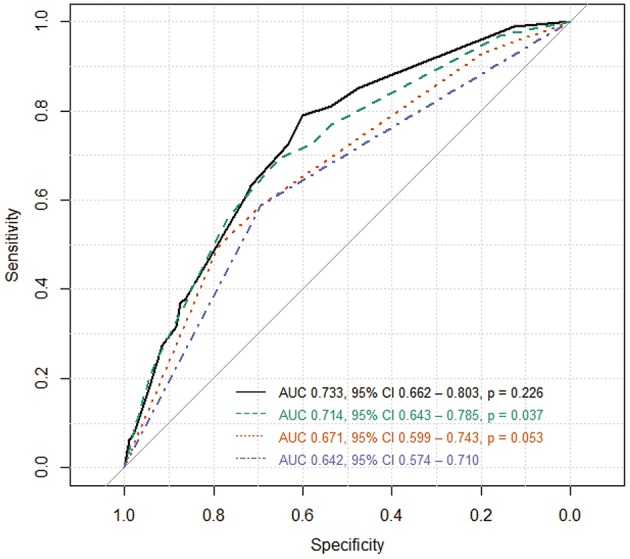
Receiver operating characteristic (ROC) curve. The accuracy of differentiating SCAS from ACAS was measured using several combination of factors associated SCAS: ROC curve of DWMH alone (blue line), DWMH and absent plaque calcification (orange line), DWMH, absent plaque calcification, and GRE HV sign (green line), and DWMH, absent plaque calcification, GRE HV sign, and increased cerebral blood volume (black line). The combination of all four factors (black line) yielded the best accuracy. AUC indicates area under the ROC curve; CI, confidence interval; p, *p*-value comparing the two AUCs.

The accuracy of differentiating SCAS from ACAS was assessed using several combination of factors associated with SCAS (Figure 2): ROC curve of DWMH alone (blue line), DWMH and absent plaque calcification (orange line), DWMH, absent plaque calcification, and GRE HV sign (green line), and DWMH, absent plaque calcification, GRE HV sign, and increased cerebral blood volume (black line). The combination of all four factors (black line) yielded the best accuracy to classify SCAS (AUC = 0.733, 95% CI 0.662–0.803; Figure 2).

## Discussion

The major finding of our study is that in addition to the conventional risk factors for SCAS, vascular imaging and PWI parameters may provide important information about uncompensated hypoperfusion associated with SCAS. The absence of calcification of the plaque, more extensive DWMH, the presence of HV in GRE, and increased CBV were independently associated with SCAS. The combination of these factors better classified SCAS from ACAS than each factor. Multimodal imaging may therefore provide additional risk stratification in ACAS patients.

When present in ACAS, the markers of hypoperfusion that were associated with SCAS likely indicate that brain perfusion status is approaching a critical threshold for symptomatic ischemia.

In our retrospective cohorts who underwent carotid revascularization, patients had different indications for the revascularization and thus differences between baseline characteristics. Although the prospective observation of ACAS patients would yield the most unbiased results, such study requires a large number of samples and long observation time since the rate of primary outcome events (symptomatic stroke or transient ischemic attack) is low in patients with ACAS. To overcome our study design, we used the propensity score matching to approximate randomization by using baseline variables and identify matched pairs.

The two major mechanisms of transient ischemic attack or ischemic stroke in carotid stenosis are artery-to-artery embolism from an unstable plaque and low perfusion due to severe stenosis and poor collaterals. Previous studies suggested the nature of the plaque as a predictor of the risk of stroke in patients with ACAS. Plaque ulceration, echolucent plaque, and microembolic signals were suggested as risk factors of ischemic stroke in ACAS patients (20–22). Using ultrasonography, plaque instability indicated by echolucent, hypoechoic plaque (23), or heterogeneous echocontrast (24). In addition, patients with a juxtaluminal hypoechoic area of >8 mm in the plaque had a high occurrence rate of ischemic stroke (25). Plaque ulceration or intraplaque hemorrhage on magnetic resonance carotid artery imaging indicate instability of the plaque (26, 27). In our study, there were no significant associations between in plaque characteristics on in carotid duplex sonography and symptomatic status. However, after propensity matching, SCAS was associated with longer plaque length in univariable analysis and calcification of the plaque was more frequent in the ACAS group in multivariable analysis. Longer plaque length was associated with greater risk of ischemic stroke either due to small emboli from the plaque or hypoperfusion (28). Plaque calcification has been suggested as an indicator of greater plaque stability and lower embolic risk. Plaque composition analysis using CT angiography revealed calcification proportion was inversely associated with plaque ulceration (29). There is also evidence that patients with less calcification of the plaque on carotid computed tomography had more stroke events (30).

In previous MRI-based carotid stenosis studies, silent infarcts on FLAIR MRI, contralateral carotid artery stenosis on brain MRA and increased mean transit time and decreased CBF on PWI were shown to predict stroke events in patients with ACAS (29–31). In our SCAS group, DWMH, HVS in FLAIR and HV in GRE were detected more often on brain MRI. Previous studies have shown that WMH is associated with an increased risk of cerebrovascular events (32, 33), and is a predictor of stroke due to atherosclerosis (34).

DWMH represents the area of chronic hypoperfusion in the brain. The significantly higher frequency of DWMH in the SCAS group indicates a decreased threshold for ischemic events. HVS in FLAIR has usually been described in hyperacute strokes with arterial occlusion and indicate collateral vessels (17). Between the two groups, the SCAS group showed a higher rate of HVS in FLAIR than the ACAS group, but HVS was not independently associated with SCAS in the multivariable analysis. HV in GRE has been used to predict the state of cerebral hypoperfusion and is thought to represent increased oxygen extraction fraction (35). In our study, CBV was increased in the SCAS group. Increased CBV by compensatory vasodilatation can develop in patients with chronic hemodynamic insufficiency, which in turn acts as a risk factor for further ischemic events if the compensation fails to properly respond.

There are some limitations of this study. First, the study was retrospective and there was no imaging of patients with carotid lesions prior to them becoming symptomatic. Brain MRI and PWI parameters were obtained during or after the acute ischemic period in the SCAS group: MR including PWI was acquired within about 6 days, [Median 6.0 days, interquartile range (IQR) 1.0–17.0 days]. The observed CBV abnormalities may reflect chronic stenosis rather than acute stroke. Second, we included patients who underwent carotid artery revascularization procedures, so we did not include all patients who received medical treatment. In accordance with AHA/ASA guidelines, symptomatic patients with ≥50% stenosis were considered for carotid artery revascularization procedures (10). In this study, all patients had more than moderate stenosis, so our results of this study might indicate specific risk factors for patients who need revascularization procedures rather than the best medical treatment. In addition, although differences in baseline characteristics between groups were balanced using the propensity score matching, unmeasured confounding factors by variables not available in our dataset may persist. Finally, long term follow-up of moderate to severe ACAS with quantitative analysis of WMH, HVS in FLAIR, HV in GRE, and PWI parameters is necessary to validate the predictive value of the multimodal carotid imaging approach for SCAS.

Strengths of our study include the use of propensity score matching to reduce the impact of selection bias. The recent treatment guidelines for ACAS emphasize the importance of best medical treatment with strict control of risk factors (36). However, some patients with ACAS still had ischemic stroke events, despite receiving the best medical treatment. Prediction for SCAS is important to select candidates for carotid endarterectomy and carotid artery stenting. The DWMH, HVS in FLAIR, HV in GRE, and increased CBV were shown association with SCAS independently. In addition, a calcified plaque revealed a low risk for SCAS. DWMH, FLAIR HVS sign, and HV in GRE can provide information on tissue perfusion without performing PWI.

## Conclusions

Multimodal imaging including computed tomography angiography, conventional MRI, and PWI may be useful in the identification of patients at high risk of ischemic stroke among patients with ACAS. Further validation with prospective patient registration and long term follow-up is required to prove the predictive value of these parameters.

## Data Availability

All relevant data are within the article. Raw data can be obtained by contacting the corresponding author (kimgm@skku.edu)

## Ethics Statement

The Institutional Review Board of Samsung Medical Center approved this study and each patient provided written informed consent.

## Author Contributions

MP and SK: study design, data analysis, statistical analysis, and manuscript drafting. ML data analysis. KK, PJ, YP, YK, DK, OB, CC, and KL: data acquisition and data analysis. GK: study concept and design, data acquisition, data analysis, and manuscript correction.

### Conflict of Interest Statement

The authors declare that the research was conducted in the absence of any commercial or financial relationships that could be construed as a potential conflict of interest.

## Abbreviations

ACAS: asymptomatic carotid artery stenosis
SCAS: symptomatic carotid artery stenosis
MRI: magnetic resonance imaging
MRA: magnetic resonance angiography
PWI: perfusion weighted imaging
DWMH: deep white matter hyperintensity
PWMH: periventricular white matter hyperintensity
HVS: hyperintense vessel signal
HV: hypointense vessels
CBV: cerebral blood volume
TTP: delayed time to peak
VIF: variance inflation factor
AUC: area under ROC
CI: confidence interval.
