# Supplementary material for: Identification of High Risk Carotid Artery Stenosis: A Multimodal Vascular and Perfusion Imaging Study
Source: Front Neurol. 2019 Jul 16;10:765. doi: 10.3389/fneur.2019.00765 (PMC6647800; doi:10.3389/fneur.2019.00765)
Supplement: Supplementary file 1 [file Image_1.pdf]

Figure S1. Selection of patients

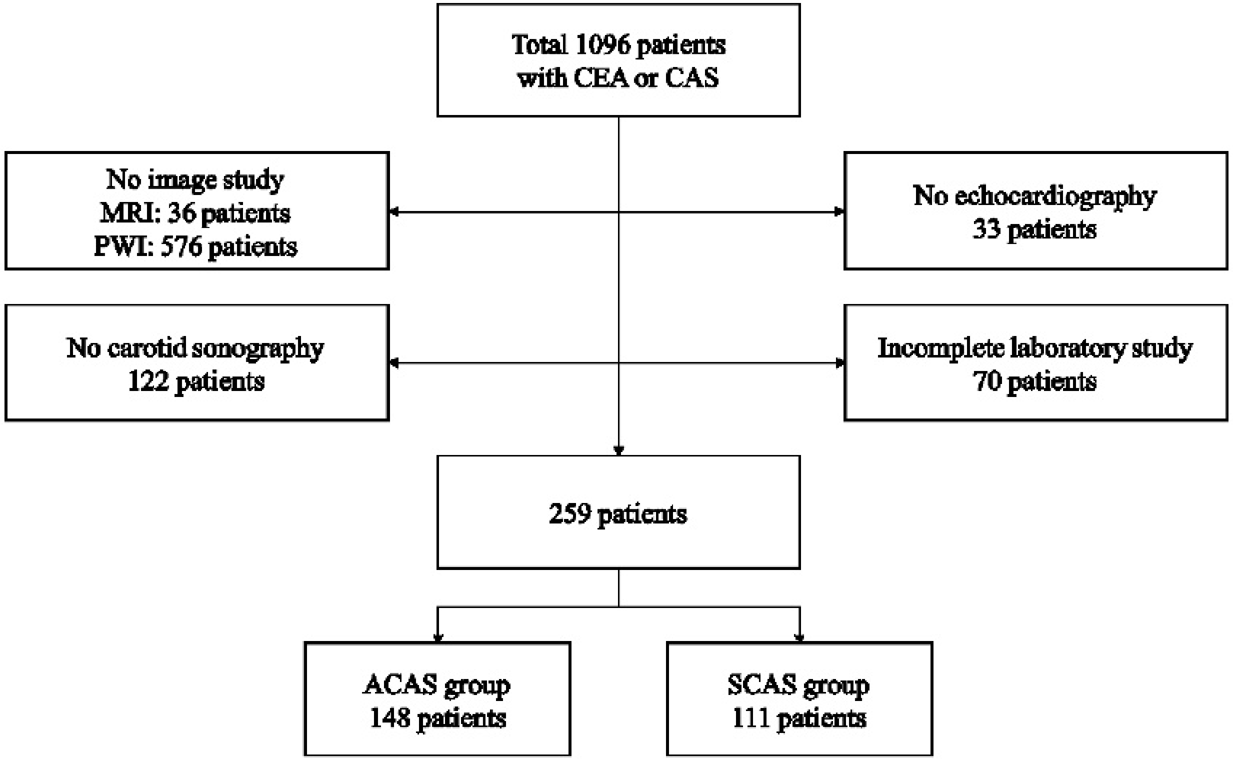

CEA, carotid endarterectomy; CAS, carotid artery stenting; MRI, magnetic resonance imaging; PWI, perfusion
